# The Protective Effect of Neighbourhood Collective Efficacy On Family Violence and Youth Antisocial Behaviour in Two South Korean Prospective Longitudinal Cohorts

**DOI:** 10.1007/s10802-021-00869-y

**Published:** 2021-09-22

**Authors:** Andreas Bauer, Gemma Hammerton, Jisu Park, Joseph Murray, Yoonsun Han, Alicia Matijasevich, Sarah L. Halligan, Graeme Fairchild

**Affiliations:** 1 Department of Psychology, University of Bath, Bath, UK; 2 Human Development and Violence Research Centre (DOVE), Federal University of Pelotas, Pelotas, Brazil; 3 Bristol Medical School, Population Health Sciences, University of Bristol, Bristol, UK; 4 MRC Integrative Epidemiology Unit, University of Bristol, Bristol, UK; 5 Institute of Social Welfare, Seoul National University, Seoul, South Korea; 6 Postgraduate Programme in Epidemiology, Federal University of Pelotas, Pelotas, Brazil; 7 Department of Social Welfare, Seoul National University, Seoul, South Korea; 8 Departamento de Medicina Preventiva, Faculdade de Medicina FMUSP, Universidade de São Paulo, São Paulo, Brasil; 9 Department of Psychiatry and Mental Health, University of Cape Town, Cape Town, South Africa

**Keywords:** Neighbourhood collective efficacy, Family violence, Child abuse, Domestic violence, Antisocial behaviour, Mediation

## Abstract

Neighbourhood collective efficacy has been proposed as a protective factor against family violence and youth antisocial behaviour. However, little is known about its impact on parent and child behaviour in non-Western countries. Using data from two population-based prospective cohorts from South Korea, including primary school students aged 10–12 years (*N* = 2844) and secondary school students aged 15–17 years (*N* = 3449), we examined the interplay between collective efficacy, family violence, and youth antisocial behaviour, and whether effects vary by SES. In a first series of models, in both samples, higher levels of collective efficacy were associated with lower levels of family violence, whereas higher levels of family violence were associated with higher levels of youth antisocial behaviour. There was no direct effect of collective efficacy on youth antisocial behaviour; however, there was an indirect effect via family violence. Although these effects were more pronounced in low SES children, there was no evidence of moderation by SES. In a second series of models, in primary school students, collective efficacy was not associated with youth antisocial behaviour. However, there was a direct effect of collective efficacy on family violence, even after adjusting for youth antisocial behaviour. Again, there was no evidence of moderation by SES. In secondary school students, the pattern of results was less consistent, however, again, suggesting more pronounced effects of collective efficacy in low SES children. The findings suggest that collective efficacy may influence family violence more directly, whereas youth antisocial behaviour may be affected more indirectly through the family environment.

**Supplementary Information:**

The online version contains supplementary material available at 10.1007/s10802-021-00869-y.

## Introduction

There are well-documented effects of neighbourhood structural characteristics on child and adolescent behavioural outcomes (Leventhal & Brooks-Gunn, 2000). Neighbourhood social processes have been proposed as mechanisms linking structural factors to behaviour problems among children and adolescents (Sampson et al., 2002). Thus, according to the *social disorganisation* theory, neighbourhood structural disadvantage, such as poverty and residential instability, weakens social bonds among residents, which, in turn, impedes collective neighbourhood action directed towards community problems (Shaw & McKay, 1942). As a result, residents in structurally disadvantaged neighbourhoods are less able to monitor and deter youth problem behaviour than residents of neighbourhoods with more favourable structural conditions.

Sampson et al. (1997) extended this work by developing the concept of neighbourhood collective efficacy, a measure of *social organisation*, including informal social control (i.e., the residents’ willingness to intervene) and social cohesion (i.e., mutual trust among neighbours). In a landmark study, Sampson et al. (1997) demonstrated that collective efficacy is a key factor in explaining the association between neighbourhood structural factors and community violence. More specifically, collective efficacy largely mediated the associations of concentrated disadvantage and residential instability with violent crime. Furthermore, a meta-analysis identified low collective efficacy as one of the strongest neighbourhood-level predictors of crime (Pratt & Cullen, 2005). Thus, collective efficacy has been proposed as a mechanism through which neighbourhood structural characteristics influence aggressive and antisocial behaviour in young people (Leventhal & Brooks-Gunn, 2000). For example, using data from the Environmental Risk Longitudinal Twin study, a nationally representative cohort from the UK, Odgers et al. (2009) examined the association between neighbourhood collective efficacy and developmental trajectories of antisocial behaviour from ages 5–10 years. In deprived, but not affluent, neighbourhoods, collective efficacy was negatively associated with children’s antisocial behaviour at school entry, even after adjusting for adverse family characteristics, such as family violence.

Similar to aggressive and antisocial behaviour in young people, collective efficacy has been proposed as a mechanism linking neighbourhood structural characteristics to child maltreatment (Coulton et al., 2007; Zielinski & Bradshaw, 2006). Thus, collective efficacy may provide community and social support to families, especially in structurally disadvantaged neighbourhoods, which, in turn, may decrease the use of harsh and abusive parenting strategies (Coulton et al., 2007; Molnar et al., 2016; Zielinski & Bradshaw, 2006). Furthermore, collective efficacy has been shown to be associated with a decrease in domestic violence (Beyer et al., 2015; Jackson, 2016; Pinchevsky & Wright, 2012; Wright & Benson, 2010), which often co-occurs with child abuse and neglect (Hamby et al., 2010), indicating the clustering of different forms of family violence.

Although parent characteristics have been proposed as pathways through which neighbourhood effects are transferred to children and adolescents (Leventhal & Brooks-Gunn, 2000; Sampson & Laub, 1993), given the well-documented bidirectional effects between parent and child behaviour (Pinquart, 2017), the reverse may equally apply – child characteristics as pathways through which neighbourhood effects are transferred to parents. For example, while child abuse is considered a key risk factor for aggressive and antisocial behaviour in children (Braga et al., 2017; Wilson et al., 2009), child externalising problems have been shown to elicit more harsh and abusive parenting (Pinquart, 2017), indicating a reciprocal relationship. According to this logic, decreases in child externalising symptoms, as a result of, for example, neighbourhood intervention, would be associated with decreases in harsh parenting.

In sum, collectively efficacy has been proposed to exert protective effects on both youth antisocial behaviour and family violence, which, in turn, show bidirectional associations. However, these potential mediating pathways have not been investigated systematically. In the Fragile Families and Child Well-Being Study, a nationally representative US birth cohort, low levels of neighbourhood collective efficacy and high levels of corporal punishment were independently associated with externalising problems among children aged 3–5 years (Ma, 2016; Ma & Grogan-Kaylor, 2017). However, corporal punishment did not mediate the association between collective efficacy and child externalising problems. Thus, while harsh parenting as a proximal mechanism through which neighbourhood collective efficacy may influence child externalising problems has received some attention, the alternative pathway of child behavioural problems as a mediator between collective efficacy and family violence has been largely ignored.

The effects of neighbourhood- and family-level factors may vary across development. For example, harsh and abusive parenting may have more detrimental effects on aggressive and antisocial behaviour for younger compared to older children, possibly due to the relative rarity of corporal punishment in adolescence or the greater influence of factors outside the family environment, such as peers, for older children (Gershoff, 2002). The latter is particularly important when considering developmental differences in neighbourhood effects. As parents increase the level of autonomy and the time to engage in activities outside the home environment for children with increasing age (Veitch et al., 2006), older children may have more interactions with residents from the neighbourhood. Thus, it has been proposed that direct neighbourhood influences may be stronger in adolescence when time spent outside increases, whereas, in childhood, effects may be more indirect, i.e., mainly mediated through the family environment (Leventhal & Brooks-Gunn, 2000). According to this logic, we may expect the indirect pathway of collective efficacy on youth antisocial behaviour through family violence to be stronger in younger children, and the direct effect of collective efficacy on youth antisocial behaviour to be stronger in older children. Similarly, the indirect effect of collective efficacy on family violence via youth antisocial behaviour would be stronger in older children.

To date, studies examining the interplay between neighbourhood collective efficacy, family violence, and antisocial behaviour in children and adolescents have mainly been conducted in Western countries (Jaffee et al., 2007; Ma, 2016; Ma & Grogan-Kaylor, 2017; Odgers et al., 2009; Wilkinson et al., 2019; Yonas et al., 2010). Little is known about the generalisability of findings to non-Western countries, where cultural differences may influence neighbourhood relationships and shared expectations of informal social control towards community problems. For example, Yoshizawa et al. (2020) found no effects of neighbourhood collective efficacy on youth antisocial behaviour across three different Asian countries.

To summarise, there is evidence for protective effects of neighbourhood collective efficacy on youth antisocial behaviour and family violence. However, the pathways through which collective efficacy influences parent and child behaviour remain poorly understood. More specifically, although there are well-established bidirectional effects between harsh and abusive parenting and child externalising problems (Pinquart, 2017), studies have been limited to family violence as a mediator of the association between collective efficacy and antisocial behaviour, as opposed to the reverse association of antisocial behaviour as a mediator between collective efficacy and family violence. Furthermore, these studies have been limited to early childhood, as opposed to other developmental periods, and focused on corporal punishment, rather than more severe or other forms of family violence (Ma, 2016; Ma & Grogan-Kaylor, 2017). In addition, most studies have been limited to high-risk (i.e., low SES) samples (e.g., the Project on Human Development in Chicago Neighbourhoods), such as inner-city neighbourhoods in the US, with some studies suggesting that the protective effect of collective efficacy may be limited to these settings (Odgers et al., 2009). Thus, it is particularly important to investigate whether effects of collective efficacy vary by SES. Finally, the vast majority of studies examining the protective effects of collective efficacy have been conducted in Western countries, and the generalisability of these findings to other cultural contexts is unknown.

To address these gaps in the literature, we examined longitudinal associations between neighbourhood collective efficacy, family violence, and youth antisocial behaviour, using two nationally representative cohorts from South Korea. These included primary school students followed from age 10 to 12 years and secondary school students followed through ages 15 to 17 years, which enabled us to examine whether direct and indirect effects would be replicated across age groups. The main objectives of the present study were: (i) to examine whether higher levels of collective efficacy are associated with decreases in both youth antisocial behaviour and family violence over time; (ii) to test whether there are indirect effects of collective efficacy on youth antisocial behaviour through family violence and on family violence via youth antisocial behaviour; (iii) to examine whether there is evidence of remaining direct effects of collective efficacy on youth antisocial behaviour (after adjusting for family violence) and family violence (after adjusting for youth antisocial behaviour); (iv) to examine whether these associations are evident for both younger and older children; and (v) to investigate whether these effects vary by SES. Based on previous research (Odgers et al., 2009), we predicted that the effects of collective efficacy would be more pronounced in children from low, as compared to medium–high, SES backgrounds.

## Methods

### Participants

The present study used data from the Korean Youth Panel Survey collected by the National Youth Policy Institute (NYPI), including two population-based prospective cohorts. The first survey was conducted from 2003 to 2008, including six annual waves from the 2nd year of secondary school to one year after graduation. All second-year junior high school students (*N* = 618,100) nationwide (except Jeju Island) were eligible for inclusion. A total of 3697 students were selected based on stratified multi-stage cluster sampling at the regional, school, and classroom levels. More precisely, at baseline, one classroom in each school was randomly selected based on 15 administrative districts in South Korea. The survey data comprised 3449 (93%; 50% boys) students and their parents at age 14 years (i.e., baseline). Children and their parents were assessed again at ages 15 (92%), 16 (91%), 17 (91%), 18 (86%), and 19 (82%) years. This cohort is hereafter referred to as the *secondary school* sample. The second survey was conducted from 2004 to 2008, including five annual waves from the 4th grade of primary school to the 2nd year of secondary school. All fourth-year elementary school students (*N* = 630,694) nationwide (except Jeju Island) were eligible for inclusion. A total of 2949 students were selected using the same sampling method as for the secondary school sample. The survey data comprised 2844 (96%; 54% boys) students and their parents at age 10 years (i.e., baseline). Children and their parents were assessed again at ages 11 (95%), 12 (94%), 13 (88%), and 14 (86%) years. This cohort is hereafter referred to as the *primary school* sample. We used waves 1–3 (i.e., ages 10–12 years) in the primary school sample, whereas, in the secondary school sample, we used waves 2–4 (i.e., ages 15–17 years), as measures of neighbourhood collective efficacy were not available at baseline. Written informed consent was obtained from children and their parents in both cohorts. More precisely, interviewers sent consent forms to schools prior to the survey, and they collected self-report data from children who agreed to participate. Next, parents were invited by mail to participate, and were subsequently interviewed by telephone. Children’s data were excluded when their parents refused to participate in the study. In subsequent waves, children were individually contacted to conduct face-to-face interviews and parents were interviewed by telephone. Further details on the two cohorts are available in English on the NYPI website (www.nypi.re.kr).

### Measures

#### Neighbourhood Collective Efficacy

Collective efficacy was measured at wave 1 (age 10 years) in the primary school sample and wave 2 (age 15 years) in the secondary school sample. Children were asked whether neighbours: (1) have close relationships with each other, (2) trust each other, (3) scold them if they smoke or drink in the neighbourhood, and (4) intervene or report to the police if they are assaulted in the neighbourhood, and whether they (5) let neighbours know if friends smoke or drink in the neighbourhood and (6) intervene or report to the police if friends are assaulted in the neighbourhood. The six items were rated on a 5-point scale, from 0 ‘*very untrue*’ *to* 4 ‘*very true*’. Internal reliabilities were acceptable with ω = 0.67 and ω = 0.80 for the primary and secondary school samples, respectively.

#### Family Violence

Domestic violence and child abuse were measured at waves 2 (age 11 years) and 3 (age 12 years) in the primary school sample and waves 3 (age 16 years) and 4 (age 17 years) in the secondary school sample. Children were asked whether they frequently see: (1) their parents verbally abuse each other or (2) one parent beat the other one, and whether they are often (3) verbally abused or (4) severely beaten by parents. The first two items were used to assess domestic violence, while the latter two items were used to assess child abuse. The four items were rated on a 5-point scale, from 0 ‘*very untrue*’ to 4 ‘*very true*’, and were used to create a composite measure of family violence. The scale showed good internal reliability in the primary school sample at waves 2 (ω = 0.78) and 3 (ω = 0.82) and in the secondary school sample at waves 3 (ω = 0.80) and 4 (ω = 0.86).

#### Youth Antisocial Behaviour

Children were asked about antisocial and aggressive behaviours at waves 2 (age 11 years) and 3 (age 12 years) in the primary school sample and waves 3 (age 16 years) and 4 (age 17 years) in the secondary school sample. Overall, 14 items were used in the primary school sample and 11 items were used in the secondary school sample, of which 10 were identical across cohorts, asking about behaviour problems in the past year, including unauthorised school absence, group bullying, severe teasing or banter, threatening, drinking,^1^ smoking,^2^ severely beating others, robbing, stealing, and running away. In addition, children in the primary school sample were asked whether they engaged in the following four problem behaviours: fare evasion, shouting at their teacher, cheating on an exam, and misappropriating expenses for school supplies. Children in the secondary school sample were additionally asked whether they had engaged in a gang fight. All items were coded as either 0 ‘*no*’ or 1 ‘*yes*’. Analyses were based on 14 and 11 items for the primary and secondary school samples, respectively. The scales showed excellent internal reliability in the primary school sample at waves 2 (ω = 0.94) and 3 (ω = 0.94) and in the secondary school sample at waves 3 (ω = 0.95) and 4 (ω = 0.95).

#### Covariates

We included child sex, family composition, and indicators of income and education, each of which have been identified as risk factors for family violence and child antisocial behaviour (Piotrowska et al., 2015; Stith et al., 2009). Information on all covariates except child sex was collected by parent report at wave 1 in both samples. Child sex was coded as 0 ‘*female*’ or 1 ‘*male*’. Family composition was coded as 0 ‘*living with biological father and mother*’ or 1 ‘*other*’. House ownership was coded as 0 ‘*own house*’ or 1 ‘*other*’. Maternal and paternal education were coded as 0 ‘*no schooling*’, 1 ‘*elementary school*’, 2 ‘*middle school*’, 3 ‘*high school*’, 4 ‘*junior college*’, 5 ‘*college/university*’, 6 ‘*master’s degree*’, and 7 ‘*PhD*’. Average monthly family income was used as a continuous variable, measured in Korean Won (₩), with ₩1035 equating approximately to USD 1 in 2004 when the studies commenced.

### Analysis Strategy

For both samples, we specified two fully latent structural regression models to examine the associations between neighbourhood collective efficacy, family violence, and youth antisocial behaviour.

**Fig. 1:**
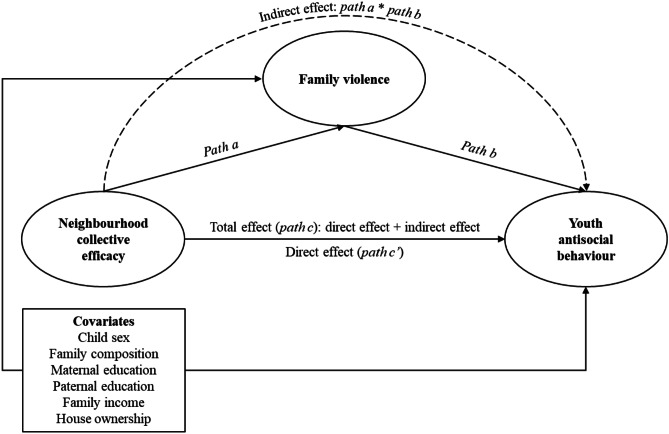
Hypothesised model with family violence as a mediator of the association between neighbourhood collective efficacy and youth antisocial behaviour

**Fig. 2:**
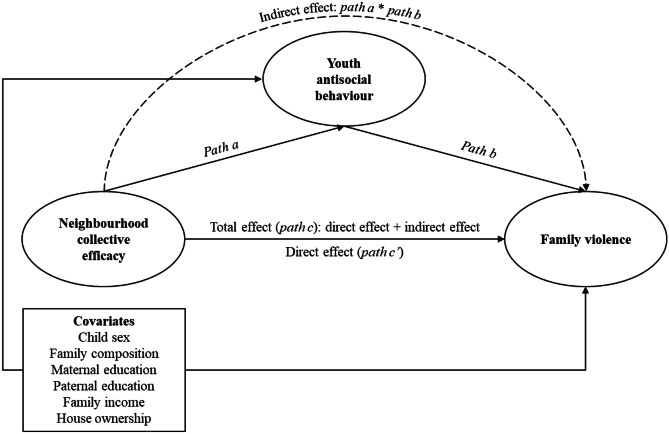
Hypothesised model with youth antisocial behaviour as a mediator of the association between neighbourhood collective efficacy and family violence

In a first series of models, the *structural* parts represented the hypotheses that: (i) collective efficacy has an effect on family violence; (ii) collective efficacy and family violence each have effects on youth antisocial behaviour; and (iii) collective efficacy also affects youth antisocial behaviour indirectly through family violence (i.e., family violence is a mediator in the association between collective efficacy and youth antisocial behaviour; see Fig. 1 for a schematic diagram of hypothesised relations). In a second series of models, we tested for reversed associations, specifically whether youth antisocial behaviour might influence family violence. Here, the *structural* parts represented the hypotheses that: (i) collective efficacy has an effect on youth antisocial behaviour; (ii) collective efficacy and youth antisocial behaviour each have effects on family violence; and (iii) collective efficacy also affects family violence indirectly through youth antisocial behaviour (see Fig. 2 for a schematic diagram of hypothesised relations).

The *measurement* part of each model featured three factors, including collective efficacy with six indicators, family violence with four indicators, and youth antisocial behaviour with 14 and 11 indicators for the primary and secondary school samples, respectively.

First, each measurement model was re-specified as a confirmatory factor analysis (CFA) model with correlated factors. The following indices were used to evaluate model fit: Comparative Fit Index (CFI) and Tucker-Lewis Index (TLI), Root Mean Square Error of Approximation (RMSEA), and Standardised Root Mean Residual (SRMR), with values of ≥ 0.95, ≤ 0.06, and ≤ 0.08, respectively, indicating good model fit (Hu & Bentler, 1999). The chi-square statistic was not used to evaluate model fit as models based on large samples are too readily rejected.

Second, we examined hypotheses about direct, indirect, and total effects among latent variables in each structural model. In a first set of models, we tested whether collective efficacy is negatively associated with family violence (i.e., *path a*); whether family violence is positively associated with youth antisocial behaviour after adjusting for collective efficacy (i.e., *path b*); and whether collective efficacy is negatively associated with youth antisocial behaviour (i.e., *path c* or *total effect*). We also tested whether the association between collective efficacy and youth antisocial behaviour holds when adjusting for family violence (i.e., *path c’* or *direct effect*), and whether family violence mediates the association between collective efficacy and youth antisocial behaviour (i.e., *indirect effect*; see Fig. 1). In a second set of models, we switched the mediator and the outcome, to test the alternative hypothesis that higher levels of collective efficacy are associated with decreased levels of youth antisocial behaviour, which, in turn, are associated with a decrease in family violence (see Fig. 2).

We followed Hayes’ (2018) approach to mediation, and estimated indirect effects even if *paths a* and *b* were non-significant, as well as in the absence of a significant total effect (*path c*). Indirect effects were estimated using the product of coefficient strategy with 1000 bootstrap samples and bias-corrected 95% confidence intervals (Williams & MacKinnon, 2008). Wald’s test was used for determining whether path coefficients differed between low and medium–high SES. Children from families with a monthly income in the lowest quintile were classified as low SES (primary school sample: ≤ ₩2,000,000, approximately USD 1,932; secondary school sample: ≤ ₩1,800,000, approximately USD 1,740).^3^ All models were adjusted for child sex, family composition, house ownership, maternal and paternal education, and family income. CFA and mediation analysis were performed in Mplus, Version 8.1 (Muthén & Muthén, 2017). All other analyses were performed in RStudio, Version 1.1.447 (RStudio Team, 2016).

#### Missing Data

Using full information maximum likelihood, in the primary school sample, the CFAs were based on 2844 participants (i.e., full baseline sample) and the mediation analyses after adjusting for covariates were based on 2667 (94%) participants. In the secondary school sample, the CFAs were based on 3346 (97%) participants and the mediation analyses after adjusting for covariates were based on 3059 (89%) participants, using full information maximum likelihood (Online Resource 1 presents a flow chart of retention for each cohort). Those included in the mediation analyses were less likely to live with both biological parents compared to those from the baseline samples (primary school sample: OR 0.73, 95% CI 0.55–0.97; secondary school sample: OR 0.75, 95% CI 0.61–0.93) (see Online Resource 2 for all comparisons between the baseline samples and those included in the mediation analyses).

## Results

### Descriptive Statistics

In both samples, over 90% of children lived with both biological parents, around two thirds of families owned their own homes, and about 40% of children had at least one parent with a university degree. The average monthly income was approximately ₩3,000,000 (around USD 2,899 at the time of data collection) in both samples. Table 1 shows the full sample characteristics for each cohort and all sample comparisons. Compared to the secondary school sample, the primary school sample had higher levels of parental education, lower levels of home ownership, children were more likely to live with both biological parents, and children reported higher levels of neighbourhood collective efficacy, and lower levels of family violence and antisocial behaviour (see Table 1 for full details and Table 2 for the correlation matrices for the primary school sample and secondary school sample; sample proportions for all antisocial behaviour items are presented in Online Resource 3).

### Family Violence as a Mediator of the Association Between Neighbourhood Collective Efficacy and Youth Antisocial Behaviour

The measurement models for both the primary school (CFI = 0.91; TLI = 0.90; RMSEA = 0.03; SRMR = 0.06) and secondary school (CFI = 0.94; TLI = 0.93; RMSEA = 0.03; SRMR = 0.07) samples indicated acceptable model fit (Online Resource 4 presents the factor loadings across samples).

Table 3 shows standardised path estimates after adjusting for covariates for the total sample and separated by SES across cohorts (see Fig. 1 for a schematic diagram). In the primary school sample, for the total sample, higher levels of collective efficacy were associated with decreases in family violence, and higher levels of family violence were associated with increases in youth antisocial behaviour. There was no evidence of a direct or total effect of collective efficacy on youth antisocial behaviour. However, there was a small indirect effect of collective efficacy on youth antisocial behaviour via family violence (see Table 3). There was no evidence that the pattern of effects differed across SES groups (Wald’s test *p*-values ranging between 0.19 and 0.99). However, overall, findings were somewhat more pronounced in the low SES group with the total effect of collective efficacy on youth antisocial behaviour and the indirect pathway via family violence being statistically significant in the low, but not the medium-high, SES group (see Table 3).

Similar to the primary school sample, in the secondary school sample, higher levels of collective efficacy were associated with decreases in family violence, and higher levels of family violence were associated with increases in youth antisocial behaviour. Again, there was no evidence of a direct or total effect, but there was evidence of a small indirect effect of collective efficacy on youth antisocial behaviour via family violence (see Table 3). When analyses were re-run for SES categories, there was no evidence that low and medium–high SES groups differed in terms of any direct (Wald’s test *p*-values ranging between 0.12 and 0.14) or indirect pathways (as evidenced by overlapping 95% confidence intervals; see Table 3).

### Youth Antisocial Behaviour as a Mediator of the Association Between Neighbourhood Collective Efficacy and Family Violence

The measurement models for both the primary school (CFI = 0.91; TLI = 0.90; RMSEA = 0.03; SRMR = 0.06) and secondary school (CFI = 0.93; TLI = 0.92; RMSEA = 0.03; SRMR = 0.07) samples indicated acceptable model fit (Online Resource 4 presents the factor loadings across samples).

Table 4 shows standardised path estimates after adjusting for covariates for the total sample and separated by SES across cohorts (see Fig. 2 for a schematic diagram). In the primary school sample, while collective efficacy was not associated with youth antisocial behaviour, higher levels of youth antisocial behaviour predicted increases in family violence. Furthermore, higher levels of collective efficacy were associated with decreases in family violence, even after adjusting for the effect of youth antisocial behaviour. However, there was no evidence of an indirect effect of collective efficacy on family violence via youth antisocial behaviour (see Table 4). This pattern of results were replicated when comparing low and medium–high SES groups, with no evidence of group differences (Wald’s test *p*-values ranging between 0.20 and 0.92; see Table 4).

In contrast to the primary school sample, in the secondary school sample, higher levels of collective efficacy were associated with decreases in youth antisocial behaviour, which, in turn, were associated with increases in family violence. While there was no evidence of a direct or total effect, there was evidence of a small indirect effect of collective efficacy on family violence via youth antisocial behaviour (see Table 4). When comparing SES categories, higher levels of collective efficacy were associated with lower levels of youth antisocial behaviour in low, but not medium–high, SES children (*χ*(1) = 4.63, *p* = 0.03). However, there was no evidence that SES groups differed in terms of any direct (Wald’s test *p*-values ranging between 0.20 and 0.83) or indirect pathways (as evidenced by overlapping 95% confidence intervals; see Table online4).

## Discussion

The current study examined the effects of neighbourhood collective efficacy on family violence and youth antisocial behaviour, using two nationally representative, prospective longitudinal cohorts from South Korea. In a first series of models, we examined family violence as a mediator of the association between neighbourhood collective efficacy and youth antisocial behaviour. In both the primary and secondary school samples, higher levels of collective efficacy predicted lower levels of family violence, and higher levels of family violence predicted higher levels of youth antisocial behaviour. In contrast to previous research, there was no direct effect of collective efficacy on youth antisocial behaviour. However, there was evidence of an indirect effect from collective efficacy to youth antisocial behaviour through family violence. Although there was no evidence that these effects varied according to SES, the pattern of results was more pronounced in the low SES group, with a significant total effect of collective efficacy and indirect effect via family violence in the primary school sample, which were not observed in the medium–high SES group. In a second series of models, we examined youth antisocial behaviour as a mediator of the association between neighbourhood collective efficacy and family violence. In the primary school sample, higher levels of collective efficacy were not associated with a decrease in youth antisocial behaviour. However, higher levels of collective efficacy predicted a decrease in family violence, even after adjusting for youth antisocial behaviour (i.e., collective efficacy had a direct effect on youth antisocial behaviour). There was no evidence of an indirect effect through youth antisocial behaviour or moderation by SES. Conversely, in the secondary school sample, higher levels of collective efficacy predicted a decrease in youth antisocial behaviour in low, but not medium–high, SES children, which, in turn, predicted an increase in family violence. Furthermore, there was a total effect of collective efficacy on family violence in low SES children, which, however, did not differ to medium–high SES children in direct comparison.

In contrast to previous studies (Ma, 2016; Ma & Grogan-Kaylor, 2017; Odgers et al., 2009), we found no evidence for a direct effect of collective efficacy on youth antisocial behaviour. Odgers et al. (2009) found that collective efficacy was associated with child antisocial behaviour in deprived, but not affluent, neighbourhoods. Furthermore, in the Fragile Families and Child Well-Being Study, a cohort focusing on urban children from socioeconomically disadvantaged backgrounds, there was evidence for a direct effect of collective efficacy on child externalising problems (Ma, 2016; Ma & Grogan-Kaylor, 2017). In the primary school sample, there was a total effect of collective efficacy on youth antisocial behaviour as well as an indirect effect through family violence in children from low, but not medium–high, SES backgrounds. However, when directly comparing these groups, there was no evidence of moderation by SES. Thus, while the current study may indicate a more consistent pattern of effects for low SES children, it can only provide tentative evidence for more pronounced effects in children from deprived neighbourhoods in a South Korean context.

In line with previous studies (Beyer et al., 2015; Jaffee et al., 2007; Molnar et al., 2016; Pinchevsky & Wright, 2012; Wright & Benson, 2010), higher levels of neighbourhood collective efficacy were associated with lower levels of family violence. In contrast to the pattern of effects observed for youth antisocial behaviour, there was evidence of a direct effect of collective efficacy on family violence in the primary school sample. These effects remained even after adjusting for youth antisocial behaviour, which was positively associated with family violence. While some previous studies have focused on high-risk samples, such as the Project on Human Development in Chicago Neighbourhoods (Jackson, 2016; Wright & Benson, 2010), the current study found similar results in low and medium–high SES families, which supports previous studies on the protective effect of collective efficacy on child maltreatment irrespective of structural factors (Molnar et al., 2016). In the secondary school sample, there was a less consistent pattern of results, again, merely with tentative evidence for more pronounced effects of collective efficacy in low SES families.

Previous studies have focused on cohorts based in the US and UK, and findings may not translate to other cultural contexts. The current study used two nationally representative South Korean cohorts, which included a mixture of disadvantaged and affluent families. For example, in both cohorts, around 40% of participants had at least one parent with a university degree, and over 90% of participants across samples lived with both biological parents. Thus, the current samples included a large proportion of youth from highly educated and intact families. This may explain why our findings are not in complete agreement with those obtained in samples residing in high-risk, inner-city neighbourhoods in the US. When we re-run analyses for low and medium–high SES children separately, the pattern of results were more in line with previous studies, showing larger effects for children from deprived neighbourhoods (Odgers et al., 2009). Alternatively, the absence of a direct effect of collective efficacy on youth antisocial behaviour may be explained by cultural differences. Asian cultures are viewed as more interdependent (i.e., seeing oneself as part of a greater whole), whereas American and Western European cultures are considered as more independent (i.e., seeing oneself as a distinct individual) (Markus & Kitayama, 1991). Thus, the effect of neighbourhood collective efficacy may be stronger in urban America, where social cohesion and informal social control may be considered more the exception than the rule, and where collective efficacy may provide community and social support to families that are not available elsewhere. Conversely, effects may be smaller in South Korea, where community supports are more accessible and/or already integrated into the more collectivist culture. Future research, needs to examine the constructs of collective efficacy across cultural contexts, and whether levels of collective efficacy differ across countries.

There is strong evidence for the effectiveness of parenting programmes targeting harsh and abusive parenting (Piquero et al., 2016) and perpetrators of intimate partner violence (Karakurt et al., 2019). The current findings suggest that increasing levels of neighbourhood collective efficacy may have direct effects on family violence and indirect effects on youth antisocial behaviour by reducing levels of family violence. In the US, there are promising community-based interventions, such as the Strong Communities for Children programme, which have been shown to decrease substantiated cases of child maltreatment (McDonell et al., 2015). Future research needs to ascertain whether such programmes can be translated into other cultural contexts.

According to previous research, neighbourhood influences affect adolescents more directly, whereas in childhood, these effects may operate more indirectly through proximal mechanisms, such as the family environment (Leventhal & Brooks-Gunn, 2000). However, the findings in the current study were largely comparable across younger (aged 10–12 years) and older (aged 15–17 years) children. Considering that our findings were replicated across two samples of different ages, more research is needed to examine direct and indirect pathways of neighbourhood influences, ideally using a wide age range from early childhood to late adolescence.

Key strengths of the current study include the use of two prospective, population-based cohorts from South Korea, with very high retention rates, spanning the age range of 10–17 years. Furthermore, few studies have examined the interplay between neighbourhood collective efficacy, family violence, and youth antisocial behaviour, and whether these relations vary by SES. In contrast to the vast majority of previous studies, which have used US-based samples, the current study provides novel prospective longitudinal data from a non-Western, more collectivist culture. Finally, the current study used a measure of family violence that included direct exposure to child abuse and indirect exposure through witnessing intimate partner violence, whereas previous research in this area has focused on more limited or normative forms of violence against children (e.g., corporal punishment).

The findings need to be interpreted in the context of the following limitations. First, all measures were self-report, and thus may have been subject to shared rater bias. For example, being exposed to family violence may influence young people’s perceptions of how they are treated by neighbours and the broader community. More precisely, the measurement error from using children as informants of neighbourhood influences may be correlated with the measurement error of family violence (Duncan & Raudenbush, 1999). Related to this, the current study used measures developed by the National Youth Policy Institute (South Korea), which have not been fully validated. Although our measure to assess neighbourhood collective efficacy tapped similar constructs as the scale developed by Sampson (1997) (i.e., social cohesion and informal social control), which has been widely used and is considered to be the gold standard, it was briefer and focused on alcohol use and smoking in the neighbourhood. Particularly in the primary school sample, the items related to social cohesion showed low factor loadings (see Online Resource 4). However, using a latent variable approach, we were able to minimise measurement error, and internal reliability of each latent factor and model fit of measurement models were acceptable, which should strengthen confidence in our findings. Nevertheless, future studies should use multiple sources to assess neighbourhood collective efficacy, including, for example, reports from multiple residents living in the same neighbourhood as the index child (see e.g., Odgers et al., 2009). Third, the current study included a limited number of covariates. More precisely, the association between family violence and youth antisocial behaviour may be confounded by parental mental illness and parental history of antisocial behaviour. Similarly, the association of neighbourhood collective efficacy with family violence and youth antisocial behaviour may be confounded by other neighbourhood-level variables, such as community violence, which could both reduce collective efficacy and increase family violence and youth antisocial behaviour. Fourth, we were unable to compare the results directly across school contexts due to slight differences in outcome measures. Nonetheless, in the absence of formal statistical comparisons, it is notable that effects were broadly similar across samples – with overlapping confidence intervals.

In conclusion, neighbourhood collective efficacy may affect youth antisocial behaviour more indirectly through mitigating family violence. Although, these effects were more pronounced in low SES children, there was no evidence of moderation by SES. Furthermore, neighbourhood collective efficacy may affect family violence more directly, particularly in younger children and even after adjusting for youth antisocial behaviour. Again, there was a more pronounced pattern of effects for low SES children, which, however, did not differ from the effects observed for medium–high SES children. The findings highlight the potential protective effects of collective efficacy on family violence and youth antisocial behaviour, and demonstrates the importance of proximal mechanism, such as violence in the family environment, through which neighbourhood characteristics can influence child outcomes.

**Table 1:** Sample characteristics and comparisons between the primary school sample and secondary school sample

		**Primary school**	**Secondary school**	**Comparison**
		Mean (SD) or %	Mean (SD) or %	*r* (*p*) or OR (95% CI)
**Collective efficacy** (0–24)		16.51 (4.42)	12.16 (4.58)	.43 (< .001)
**Family violence** (0–16)				
Time 2		2.22 (2.96)	2.75 (2.76)	.09 (< .001)
Time 3		2.25 (2.97)	2.86 (3.03)	.10 (< .001)
**Youth antisocial behaviour**^a^ (0–10)				
Time 2		0.41 (0.91)	0.70 (1.13)	.14 (< .001)
Time 3		0.35 (0.89)	0.77 (1.09)	.21 (< .001)
**Maternal education** (0–7)		3.50 (1.02)	3.25 (1.11)	.08 (< .001)
**Paternal education** (0–7)		3.93 (1.19)	3.74 (1.31)	.08 (< .001)
**Monthly income** (0–3000)^b^		302.14 (176.52)	299.73 (216.90)	.01 (= .63)
**Child sex** (male)		54	50	0.87 (0.78–0.96)
**House ownership** (other)^c^		38	31	0.73 (0.66–0.82)
**Family composition** (other)^d^		5	7	1.57 (1.25–1.97)

Observed, rather than latent, variables are presented.

^a^Limited to the 10 items that were identical across cohorts

^b^In units of ₩10,000 (approximately USD 10)

^c^Reference is ‘own house’

^d^Reference is ‘living with biological father and mother’

**Table 2:** Correlation matrix of all study variables in the primary school sample (*upper* triangular matrix) and the secondary school sample (*lower* triangular matrix)

		**1**	**2**	**3**	**4**	**5**	**6**	**7**	**8**	**9**	**10**	**11**
**1**	**Collective efficacy**		-.08**	-.03	-.11**	-.03	-.09**	.08**	.08**	.07**	-.11**	.01
**2**	**Family violence** (T2)	-.06**		.19**	.34**	.11**	.17**	-.09**	-.09**	-.05**	.04	.01
**3**	**Antisocial behaviour** (T2)	-.05**	.17**		.12**	.29**	.18**	-.05*	-.06**	-.02	.03	.03
**4**	**Family violence** (T3)	-.04*	.49**	.12**		.16**	.11**	-.10**	-.09**	-.04*	.03	-.03
**5**	**Antisocial behaviour** (T3)	-.04*	.12**	.55**	.13**		.08**	-.03	-.07**	-.04	.03	.09**
**6**	**Child sex** (male)	.05*	-.04	-.08**	-.01	-.14**		.00	.01	.02	.00	.05
**7**	**Maternal education**	-.04*	-.08**	-.05**	-.11**	-.05*	-.02		.68**	.38**	-.18**	-.27**
**8**	**Paternal education**	-.05*	-.08**	-.05**	-.09**	-.05**	-.02	.70**		.37**	-.20**	-.36**
**9**	**Family income**	-.02	-.08**	-.04	-.06**	-.04	-.01	.36**	.35**		-.27**	-.60**
**10**	**House ownership** (other)^a^	-.07**	.08**	.06**	.08**	.04	.04	-.14**	-.13**	-.32**		.29**
**11**	**Family composition** (other)^b^	-.08*	.09*	.08**	.09*	.07*	.03	-.30**	-.31**	-.61**	.33**	

Observed, rather than latent, variables are presented

*p < .05;** *p* < .01

^a^Reference is ‘own house

^b^Reference is ‘living with biological father and mother’

**Table 3:** Path estimates after adjusting for covariates for the total sample and separated by SES for the model examining family violence as a mediator of the association between neighbourhood collective efficacy and youth antisocial behaviour

	**Total sample**		**Medium–high SES**		**Low SES**	
	*β* (SE)	*P* or 95% CI	*β* (SE)	*P* or 95% CI	*β* (SE)	*P* or 95% CI
***Primary school***						
Collective efficacy → Family violence	**-0.11 (0.02)**	< .001	**-0.10 (0.03)**	< .01	**-0.10 (0.04)**	< .01
Family violence → Antisocial behaviour	**0.15 (0.03)**	< .001	**0.08 (0.04)**	= .04	**0.26 (0.05)**	< .001
Direct effect	-0.02 (0.03)	= .49	0.01 (0.04)	= .77	-0.09 (0.05)	= .08
Total effect	-0.04 (0.03)	= .24	0.01 (0.04)	= .91	**-0.12 (0.05)**	= .02
Indirect effect	**-0.02 (0.01)**	-0.03, -0.01	-0.01 (0.01)	-0.02, 0.00	**-0.03 (0.01)**	-0.05, -0.01
***Secondary school***						
Collective efficacy → Family violence	**-0.07 (0.02)**	< .01	**-0.06 (0.03)**	= .02	**-0.13 (0.05)**	< .01
Family violence → Antisocial behaviour	**0.18 (0.03)**	< .001	**0.17 (0.03)**	< .001	**0.24 (0.03)**	< .001
Direct effect	-0.04 (0.03)	= .15	-0.04 (0.03)	= .19	0.06 (0.03)	= .06
Total effect	-0.05 (0.03)	= .06	-0.05 (0.03)	= .12	0.03 (0.03)	= .36
Indirect effect	**-0.01 (0.01)**	-0.03, -0.01	**-0.01 (0.01)**	-0.02, -0.00	**-0.03 (0.02)**	-0.08, -0.01

All models were adjusted for child sex, family composition, house ownership, and maternal and paternal education, in addition to family income for the model using the total sample. Bold values indicate statistically significant associations at *p* < .05

*β* Standardized regression coefficient, *SE* Standard error, *95% CI* 95% confidence interval, *P* *P*-value

**Table 4:** Path estimates after adjusting for covariates for the total sample and separated by SES for the model examining youth antisocial behaviour as a mediator of the association between neighbourhood collective efficacy and family violence

	**Total sample**		**Medium–high SES**		**Low SES**	
	*β* (SE)	*P* or 95% CI	*β* (SE)	*P* or 95% CI	*β* (SE)	*P* or 95% CI
***Primary school***						
Collective efficacy → Antisocial behaviour	-0.01 (0.03)	= .70	-0.04 (0.04)	= .32	0.04 (0.05)	= .42
Antisocial behaviour → family violence	**0.15 (0.03)**	< .001	**0.13 (0.04)**	< .001	**0.17 (0.04)**	< .001
Direct effect	**-0.14 (0.02)**	< .001	**-0.11 (0.03)**	< .001	**-0.17 (0.04)**	< .001
Total effect	**-0.14 (0.02)**	< .001	**-0.12 (0.03)**	< .001	**-0.17 (0.04)**	< .001
Indirect effect	-0.00 (0.01)	-0.01, 0.01	-0.01 (0.01)	-0.02, 0.00	0.01 (0.02)	-0.03, 0.03
***Secondary school***						
Collective efficacy → Antisocial behaviour	**-0.07 (0.03)**	= .02	-0.02 (0.03)	= .55	**-0.17 (0.05)**	< .01
Antisocial behaviour → family violence	**0.17 (0.03)**	< .001	**0.16 (0.03)**	< .001	**0.23 (0.04)**	< .001
Direct effect	-0.02 (0.02)	= .34	-0.02 (0.02)	= .36	-0.06 (0.05)	= .27
Total effect	-0.03 (0.02)	= .14	-0.03 (0.02)	= .30	**-0.10 (0.05)**	= .05
Indirect effect	**-0.01 (0.01)**	-0.03, -0.00	-0.00 (0.01)	-0.02, 0.01	-0.04 (0.030)	-0.11, 0.00

All models were adjusted for child sex, family composition, house ownership, and maternal and paternal education, in addition to family income for the model using the total sample. Bold values indicate statistically significant associations at *p* < .05

*β* Standardized regression coefficient, *SE* Standard error, *95% CI* 95% confidence interval, *P* *P*-value

## Supplementary Information

Below is the link to the electronic supplementary material.

Supplementary file1 (PDF 52 KB)

Supplementary file2 (PDF 88 KB)

Supplementary file3 (PDF 37 KB)

Supplementary file4 (PDF 46 KB)

## Data Availability

All data are available in English on the South Korean National Youth Policy Institute’s website (https://www.nypi.re.kr/archive/contents/siteMain.do?srch_mu_lang=ENG).

## Footnotes

1 The legal drinking age in South Korea is 19 years.

2 The legal smoking age in South Korea is 19 years.

3 In the primary school sample, we noticed large increases in proportions for every ₩50,000, most likely as a result of rounding errors in the database. Thus, while ₩190,000 comprised 17.7% of the sample, the used cut-point of ₩200,000 already comprised 35.5% of the sample. Unfortunately, sensitivity analyses with the next lower value (₩190,000) were not feasible, as models did not converge. Thus, despite similar cut-points across both cohorts, low SES in the primary school sample might be better described as the lowest tercile as opposed to the lowest quintile.
