# Supplementary material for: The Protective Effect of Neighbourhood Collective Efficacy On Family Violence and Youth Antisocial Behaviour in Two South Korean Prospective Longitudinal Cohorts
Source: Res Child Adolesc Psychopathol. 2021 Sep 22;50(3):335–47. doi: 10.1007/s10802-021-00869-y (PMC8885499; doi:10.1007/s10802-021-00869-y)

Online Resource 1 Flow charts of included participants in the confirmatory factor analyses and mediation analyses after adjusting for covariates across samples using full information maximum likelihood

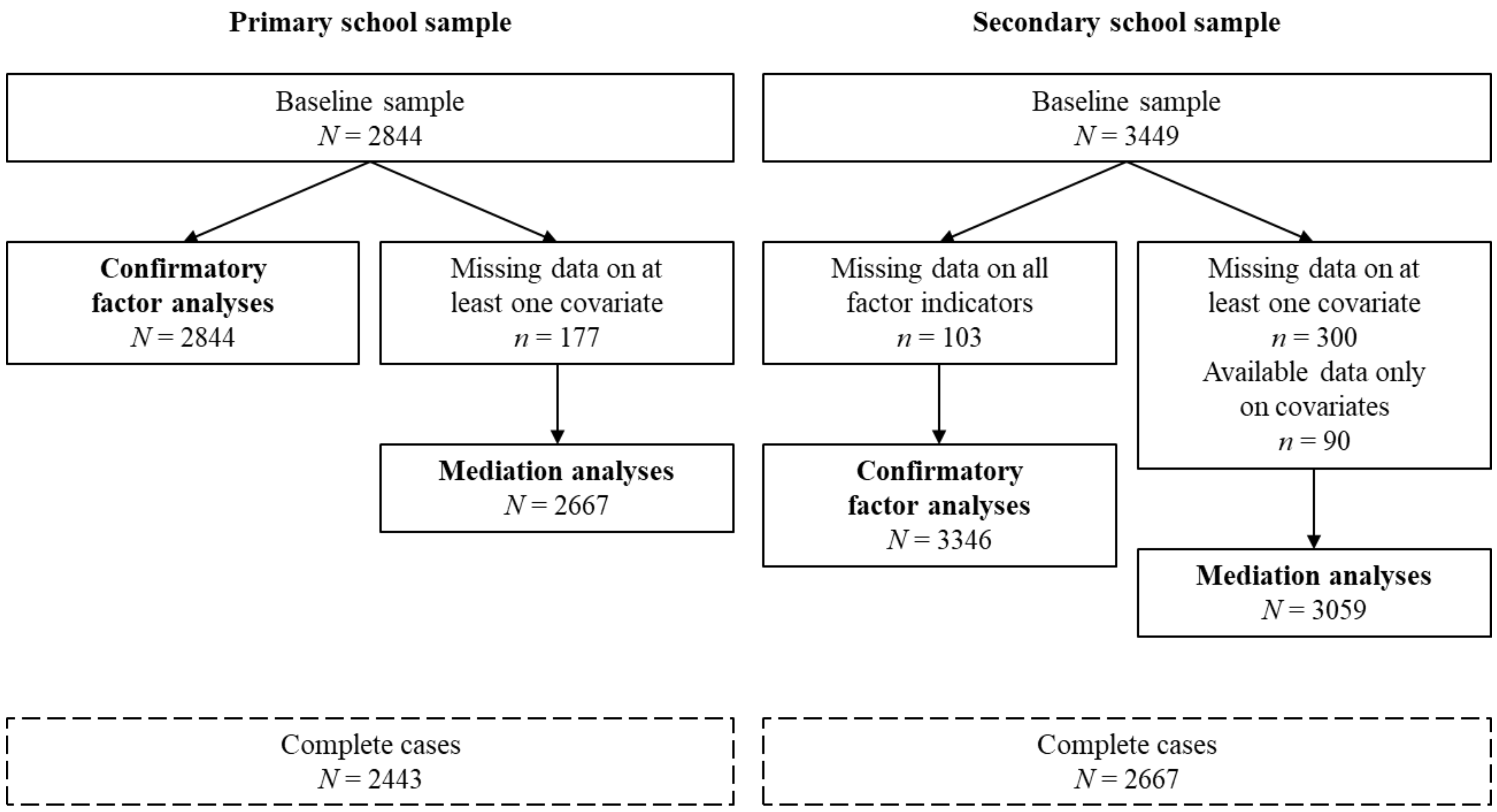

Supplement: Supplementary file 1 — Supplementary file1 (PDF 52 KB) [file 10802_2021_869_MOESM1_ESM.pdf]
