# Supplementary material for: The Protective Effect of Neighbourhood Collective Efficacy On Family Violence and Youth Antisocial Behaviour in Two South Korean Prospective Longitudinal Cohorts
Source: Res Child Adolesc Psychopathol. 2021 Sep 22;50(3):335–47. doi: 10.1007/s10802-021-00869-y (PMC8885499; doi:10.1007/s10802-021-00869-y)
Supplement: Supplementary file 2 — Supplementary file2 (PDF 88 KB) [file 10802_2021_869_MOESM2_ESM.pdf]

Online Resource 2 Comparison between the baseline sample and those included in the mediation analyses adjusted for covariates, separated by cohort

**Variables**	**Baseline**<br>Mean (SD) or %	*versus*<br>*r* (*p*) or OR (95% CI)	**Mediation**<br>Mean (SD) or %
Primary school sample			
Child's sex (male)	54	0.99 (0.89-1.10)	53
House ownership (other)[a]	38	0.98 (0.88-1.10)	37
Family composition (other)[b]	5	**0.73 (0.55-0.97)**	3
Paternal education (0-7)	3.93 (1.19)	.00 (= .78)	3.94 (1.19)
Maternal education (0-7)	3.50 (1.02)	.00 (= .91)	3.50 (1.01)
Monthly income (0-3000)[c]	302.14 (176.52)	.01 (= .49)	305.48 (176.82)
Secondary school sample			
Child's sex (male)	50	0.99 (0.89-1.09)	50
House ownership (other)[a]	31	0.96 (0.86-1.07)	30
Family composition (other)[b]	7	**0.75 (0.61-0.93)**	5
Paternal education (0-7)	4.74 (1.31)	.00 (= .91)	4.74 (1.29)
Maternal education (0-7)	4.25 (1.11)	.01 (= .66)	4.23 (1.09)
Monthly income (0-3000)[c]	299.73 (216.90)	.00 (= .92)	299.19 (214.46)

Note. Bold values indicate statistically significant group differences at $p < .05$. Pearson's correlation coefficient r and p-values were derived from independent t-tests. [a] Reference is 'own house'. [b] Reference is 'living with biological father and mother'. [c] In units of ₩10,000.
