# Supplementary material for: The Protective Effect of Neighbourhood Collective Efficacy On Family Violence and Youth Antisocial Behaviour in Two South Korean Prospective Longitudinal Cohorts
Source: Res Child Adolesc Psychopathol. 2021 Sep 22;50(3):335–47. doi: 10.1007/s10802-021-00869-y (PMC8885499; doi:10.1007/s10802-021-00869-y)
Supplement: Supplementary file 3 — Supplementary file3 (PDF 37 KB) [file 10802_2021_869_MOESM3_ESM.pdf]

Online Resource 3 Sample proportions for each item used to measure youth antisocial behaviour, separated by cohort

	**Primary school** (*N* = 2844)		**Secondary school** (*N* = 3449)	
	**Age 11** %	**Age 12** %	**Age 16** %	**Age 17** %
Unauthorised school absence	8	5	6	6
Group bullying	10	9	2	1
Severe teasing or banter	10	7	4	3
Threatening	2	2	1	1
Drinking	5	6	37	45
Smoking	1	2	12	14
Severely beating others	2	2	3	2
Robbing	1	1	1	1
Stealing	2	2	2	2
Running away	2	1	3	3
Fare evasion	2	3	N/A	
Shouting at their teacher	6	6	N/A	
Cheating on exam	7	9	N/A	
Misappropriating expenses for school supplies	10	10	N/A	
Gang fight	N/A		1	1

Note. N/A = Item not available in cohort.
