# Supplementary material for: The Protective Effect of Neighbourhood Collective Efficacy On Family Violence and Youth Antisocial Behaviour in Two South Korean Prospective Longitudinal Cohorts
Source: Res Child Adolesc Psychopathol. 2021 Sep 22;50(3):335–47. doi: 10.1007/s10802-021-00869-y (PMC8885499; doi:10.1007/s10802-021-00869-y)
Supplement: Supplementary file 4 — Supplementary file4 (PDF 46 KB) [file 10802_2021_869_MOESM4_ESM.pdf]

Online Resource 4 Standardised factor loadings of the models examining family violence as a mediator of the relationship between neighbourhood collective efficacy and youth antisocial behaviour (model 1) and the model examining youth antisocial behaviour as a mediator of the relationship between neighbourhood collective efficacy and family violence (model 2), separated by cohort

	**Primary school sample**		**Secondary school sample**	
	Model 1	Model 2	Model 1	Model 2
**Neighbourhood collective efficacy**				
My neighbours have close relationships with each other	.32	.32	.57	.58
My neighbours trust each other	.39	.39	.64	.64
Elderly neighbours will scold me if I smoke or drink in the neighbourhood	.44	.43	.72	.72
My neighbours will intervene or report to the police if I am assaulted by other kids in the neighbourhood	.62	.61	.75	.74
I will let elderly neighbours know if my friends smoke or drink in the neighbourhood	.62	.64	.53	.53
I will intervene or report to the police if my friends are assaulted in the neighbourhood	.65	.65	.54	.54
**Family violence**				
I frequently see my parents verbally abuse each other	.58	.62	.59	.69
I frequently see one of my parents beat the other one	.70	.76	.71	.79
I am often verbally abused by parents	.79	.83	.82	.83
I am often severely beaten by parents	.72	.75	.73	.78
**Youth antisocial behaviour**				
Unauthorised school absence	.56	.54	.76	.73
Group bullying	.64	.73	.65	.70
Severe teasing or banter	.76	.78	.72	.76
Threatening	.91	.74	.88	.86
Drinking	.58	.53	.74	.70
Smoking	.77	.79	.82	.78
Severely beating others	.81	.68	.81	.80
Robbing	.88	.78	.71	.89
Stealing	.76	.78	.64	.58
Running away	.67	.59	.79	.78
Fare evasion	.61	.61	N/A	N/A
Shouting at teacher	.61	.64	N/A	N/A
Cheating on exam	.66	.55	N/A	N/A
Misappropriating expenses for school supplies	.63	.53	N/A	N/A
Gang fight	N/A	N/A	.76	.78

Note. All factor loadings show $p < .001$. N/A = Item not available in cohort.
